# Impact of the Channel Length in Nanoporous Electric Double-Layer Capacitors on the Charge Transport Explored by Metal–Organic Framework Films

**DOI:** 10.1021/acsphyschemau.4c00104

**Published:** 2025-03-04

**Authors:** Yidong Liu, Abhinav Chandresh, Lars Heinke

**Affiliations:** † Institute of Functional Interfaces (IFG), Karlsruhe Institute of Technology (KIT), Hermann-von-Helmholtz-Platz 1, 76344 Eggenstein-Leopoldshafen, Germany; ‡ Physical Chemistry, Institute of Chemistry and Biochemistry, 9166 Freie Universität Berlin, Arnimallee 22, 14195 Berlin, Germany

**Keywords:** metal−organic frameworks, supercapacitors, ionic liquid, cyclic voltammetry, charge transport kinetics

## Abstract

For enhancing the performance of electric double-layer capacitors, the porous electrodes must be further optimized. While many studies on electrolyte and electrode structures enable detailed insights, the length of the pore channels of the electrode has been overlooked. Here, we use films of two-dimensional conductive metal–organic frameworks, where the film thickness (and thus the pore channel length) is rationally tuned over a wide range. Cyclic voltammetry experiments with two different electrolytes were conducted, revealing the charge transport kinetics in the porous electrodes. For the highly mobile electrolyte, the kinetics is not limited by ion transport (i.e., diffusion) even for thick films, exhibiting mainly surface-controlled kinetic behavior. In contrast, for the less mobile electrolyte, the kinetics is primarily limited by ion diffusion, and the pore channel length has a severe impact, where long channels result in strongly decreased capacitances, highlighting the importance of adjusting the channel length.

## Graphical abstract

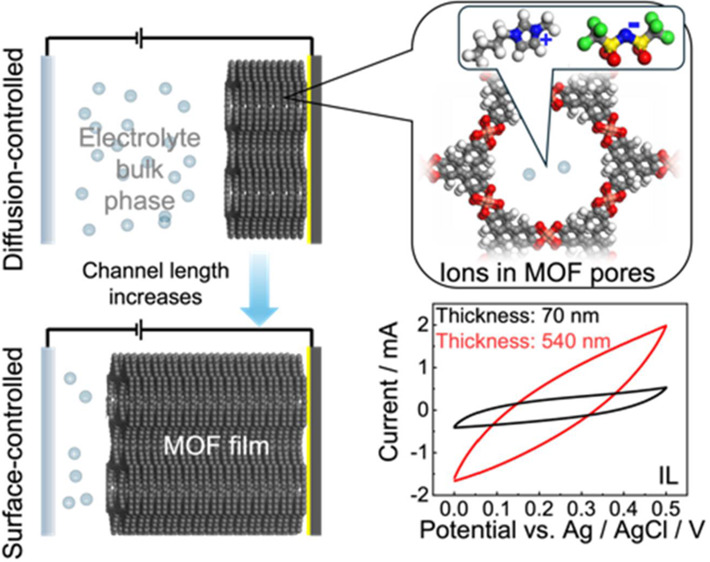

## Introduction

Electrochemical capacitors are essential components in high-power electronic devices, offering advantages such as high power density, rapid charge/discharge capabilities, and excellent cycle stability.^1,2^ Based on their charge storage mechanisms, electrochemical capacitors can be categorized into electric double-layer capacitors^3,4^ (EDLCs) and pseudocapacitors.^5^ EDLCs store energy through an interface double layer formed between the electrode and electrolyte, while pseudocapacitors rely on reversible redox reactions occurring on or near the electrode surface to generate Faradaic pseudocapacitance. The ideal electrode material for EDLCs should combine efficient charge transport with high porosity and a large surface area. Currently, nearly all active materials used in EDLCs are carbon-based, mainly activated carbon,^6^ and also carbon nanotubes^7^ and graphene.^8^ While the low cost of the carbon materials is an advantage, the difficulty (or lack) of tunability of the structure, pore size, and surface area is a disadvantage for making EDLCs with the highest performance. Metal–organic frameworks^9,10^ (MOFs), known for their high porosity, large specific surface area, and tunability, have the potential to perfectly fulfill the requirements for the electrode materials in high-performance EDLCs. However, conventional MOFs generally exhibit low electrical conductivity, limiting their competitiveness against carbon-based materials in this field. In 2012, a new class of two-dimensional MOFs was presented by linking the highly conjugated tricatecholate ligand 2,3,6,7,10,11-hexahydroxytriphenylene (HHTP) with Co­(II), Cu­(II), and Ni­(II) ions.^11^ These MOFs exhibit a conjugated structure in the horizontal plane and π–π stacking in the vertical direction, significantly enhancing their electrical conductivity with a reported conductivity of 2.1 × 10^–1^ S cm^–1^.^11^ Based on this, numerous two-dimensional conductive MOFs (2D c-MOFs) with similar structures have been designed and synthesized. Among them, Ni_3_(2,3,6,7,10,11-hexaiminotriphenylene)_2_ (Ni_3_(HITP)_2_), with a specific surface area of 630 m^2^ g^–1^ and a single-crystal conductivity as high as 50 S cm^–1^, has been applied as an electrode material for EDLCs.^12^ The resulting capacitor achieved a very high capacitance of 18 μF cm^–2^ normalized to the MOF’s specific surface area (determined by galvanostatic discharge curves). To further enhance the capacitance of 2D c-MOF-based electrodes, a smaller hexaaminobenzene (HAB) linker was utilized to construct redox-active 2D c-MOFs (Cu-HAB and Ni-HAB), showing a high specific capacitance (427 F g^–1^) and volumetric capacitance (760 F cm^–3^).^13^

Different studies have explored the mechanisms and charge transport behavior of electrolytes in 2D c-MOF-based EDLCs. Using molecular dynamics simulations, researchers studied electrolyte transport kinetics within MOF pores of various diameters.^14^ By evaluating the conductivity of ionic liquids across different pore diameters, they identified significant limitations in ion transport within the 1D channels of the 2D-MOF. These limitations indicate that the electrolyte resistance is the primary contributor to the equivalent series resistance of MOF-based EDLCs. Since the electrical conductivity of 2D c-MOFs is much higher than the ionic conductivity of confined liquids, ion transport resistance within the pores largely governs the overall device performance. In another study by combining nuclear magnetic resonance spectroscopy with density functional theory calculations, the ion adsorption behavior within the microscopic pores of Ni_3_(HITP)_2_ was investigated.^15^ The findings revealed, for tetraethylammonium tetrafluoroborate in acetonitrile, that the cations are the dominant contributors to charge storage in Ni_3_(HITP)_2_, while anions make only a minor contribution. Moreover, researchers synthesized a series of 2D c-MOFs with similar pore diameters but varying interlayer spacings.^16^ By analyzing the relationship between the peak current and the scan rate during cyclic voltammetry (CV) tests, it was observed that longer side chains hinder charge transport, thereby increasing the contribution of diffusion-controlled processes. However, this transition in the charge storage mechanism, driven by increased access to ligand-based active sites, shifted from double-layer capacitance to pseudocapacitance, leading to a progressive enhancement in the specific capacitance. By introducing functional (polar) groups into the MOF structure, the capacitive value of the pseudocapacitance and the ion mobility can also be effectively enhanced.^17^ Research has also shown that a larger specific surface area and stronger binding energy between the electrodes and electrolyte ions can enhance the specific capacitance.^18^ However, excessively strong binding energy may hinder ion desorption during charge–discharge cycles, leading to slower kinetics and reduced rate performance. So far, while the impact of the structure and functionality of the material on the EDLC performance was explored,^14–16,19–21^ the impact of the channel length, which corresponds to the thickness of the MOF film or to half the size of the MOF crystallites of a powder pressed to pellets, is not yet explored.

In this study, we systematically explored the impact of the channel length of the porous material on the capacity and charge kinetics. To this end, we select films of Cu_3_(HHTP)_2_ (Cu-CAT-1) as the model material, where the film thickness, and thus the channel length, is precisely controlled. Cu_3_(HHTP)_2_, one of the earliest synthesized 2D c-MOFs, is formed through the coordination reaction of copper ions with the HHTP ligand, as illustrated in Figure 1a. The structure of Cu_3_(HHTP)_2_ consists of stacked π-conjugated two-dimensional layers with one-dimensional cylindrical channels approximately 2 nm in diameter, which may enable efficient ion transport. The reported BET surface area is 554 m^2^ g^–1^.^22^ To explore the relationship between the MOF electrode structure and the EDLC performance, Cu_3_(HHTP)_2_ films of varying thicknesses were deposited on the substrate and used as working electrodes. Aqueous potassium chloride (KCl) solution and pure ionic liquid (IL) of type 1-butyl-3-methylimidazolium bis­(trifluoromethylsulfonyl)­imide^23,24^ ([BMIM]^+^[TFSI]^−^) were selected as electrolytes. CV measurements were performed to investigate the charge transport kinetics with the different electrolytes, and the relationship with the scan rate was explored. The data were analyzed by three different methods, which are the methods introduced by Trasatti and Petrii^25^ and Dunn and co-workers^26^ and the *b*-value analysis^27,28^ (from the Randles–Sevcik equation). We observed that the scan-rate-dependent capacitance of the supercapacitor is proportional to the MOF film thickness, indicating that the entire material (i.e., the entire pore space) is accessible for both electrolytes and the channels are not blocked. In the KCl solution, the system exhibits mainly surface-controlled kinetic behavior, where the diffusion resistance of the ions has only a minor impact on the kinetics. On the other hand, for pure IL, the kinetic is limited by the diffusion of the ions. For both electrolytes, the contribution of surface-controlled kinetics increases, and the impact of the diffusion decreases with increasing thickness of the MOF film. While the EDLC performance for KCl is equally high for thin and thick films and shows a capacitance significantly larger than that with the IL, the capacitance with the IL decreases with increasing film thickness, limiting the performance. Thus, the study shows that optimizing the film thickness allows for an enhancement of the EDLC performance, depending on the electrolyte.

**Figure 1:**
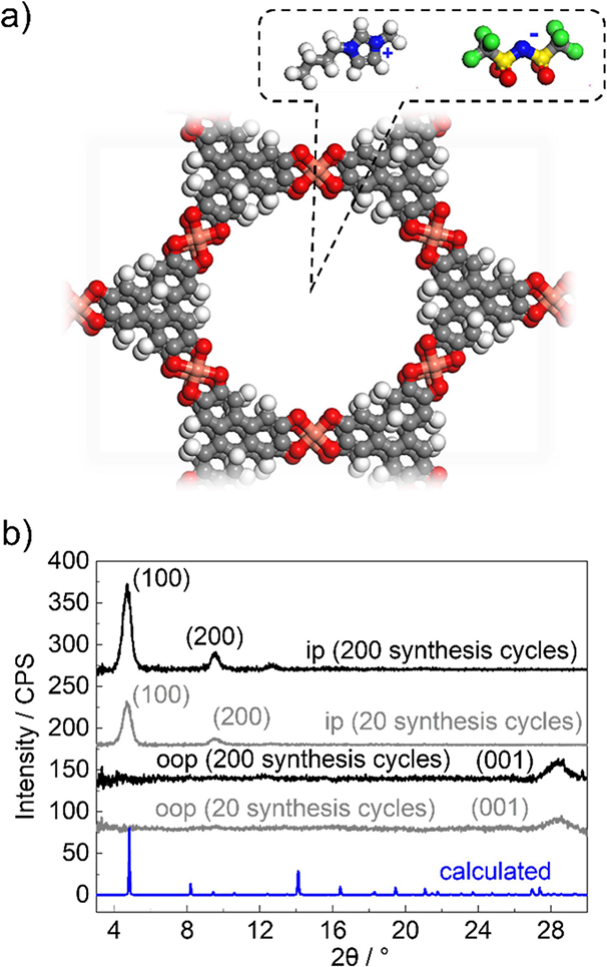
(a) Molecular structure of Cu_3_(HHTP)_2_ with the IL (type [BMIM]^+^[TFSI]^−^) electrolyte ions inside the pores. Carbon atoms are represented in gray, copper in orange, oxygen in red, nitrogen in blue, sulfur in yellow, fluorine in green, and hydrogen in white. (b) In-plane (ip) and out-of-plane (oop) XRD data of the 20-cycle and 200-cycle Cu_3_(HHTP)_2_ sample. The data are compared with the calculated XRD pattern of the targeted structure (blue). The experimentally observed diffraction peaks are labeled.

## Experimental Methods

### Materials

The chemicals, i.e., copper­(II) acetate (99.9%), 2,3,6,7,10,11-hexahydroxytriphenylene (HHTP, 98%), 11-mercapto-1-undecanol (MUD, 99%), and ethanol (99.5%), were purchased from Alfa Aesar, TCI, Sigma-Aldrich, and VWR Chemicals. They were used without further purification. Substrates for MOF growth were gold-coated silicon wafers purchased from *Georg Albert PVD coatings*.

### MOF Film Synthesis

Cu_3_(HHTP)_2_ SURMOFs were prepared in a layer-by-layer fashion by alternatively dipping the substrates in the solutions of the MOF components, i.e., ethanolic 0.1 mM copper­(ll) acetate solution and ethanolic 0.01 mM HHTP solution using a dipping robot as reported.^29^ The dipping times were 10 min for the copper acetate solution and 15 min for the HHTP solution, followed by a dipping step for 2 min with pure ethanol to remove residual reactants. The samples were prepared in 200 synthesis cycles. Afterward, the samples were immersed in ethanolic 0.01 mM HHTP solution and annealed in a 60 °C oven for 20 h, and the surface was cleaned with ethanol before use.^30^ The SURMOF substrates were MUD-modified gold-coated silicon wafers.

### Characterizations

The X-ray diffraction (XRD) data of the MOF films were recorded with a Bruker D8 ADVANCE X-ray diffractometer with Cu Kα radiation (λ = 0.154 nm). The SEM measurements were performed on a TESCAN VEGA3. To avoid charging effects, all samples were coated with a 3–4 nm thick platinum film before recording the SEM images. Electrochemical analysis was conducted using a *Gamry Reference 620* workstation, utilizing a homemade electrochemical cell configured as a three-electrode system with a Ag/AgCl reference electrode and a Pt sheet counter electrode. The CV measurements were conducted within a potential range of 0–0.3 V for KCl and 0–0.5 V for the IL, ensuring the applied voltages remained within the electrochemical window of the electrolyte and of the working electrode. A wide range of scan rates, from 0.1 to 3000 mV s^–1^, was used to capture both slow and rapid charge-transfer kinetics, providing a comprehensive assessment of the material’s electrochemical performance. The surface areal capacitance was derived from CV curves according to the equation: $C_{A}=\frac{1}{2A(\Delta V)v}\int I(\Delta V)dV$ , where *A* is the substrate area covered by MOFs.

## Results and Discussion

The fabrication of the Cu_3_(HHTP)_2_ MOF films was carried out in a layer-by-layer fashion,^31^ resulting in surface-mounted MOFs (SURMOFs).^31,32^ A dipping robot^29^ was used, which exposes the modified gold-coated silicon substrate successively to the solutions containing the MOF components, i.e., the Cu-metal nodes and the HHTP linker molecules. By controlling the number of synthesis cycles, we achieved precise regulation of the sample thickness. To ensure the reliability of the results, two independent samples were prepared. Each sample initially included Cu_3_(HHTP)_2_ synthesized through 20 cycles. After electrochemical testing, an additional 20 synthesis cycles were performed on the same sample, resulting in a 40-cycle sample, which was then retested. This synthesis–test–repeat process was continued to prepare samples with 20, 40, 60, 80, 100, 150, and 200 synthesis cycles. All samples were subsequently subjected to electrochemical analysis. Figure 1b presents the X-ray diffraction (XRD) patterns of Cu_3_(HHTP)_2_ SURMOF films after 20 and 200 synthesis cycles, revealing similar crystal structures consistent with the intended design. The in-plane (ip) XRD patterns show a high degree of crystallographic alignment, showing reflections exclusively perpendicular to the [001] direction. The out-of-plane (oop) XRD patterns display only the (001) peak, indicating that the SURMOF films grew with a preferred orientation. These findings confirm that both the 20- and 200-cycle Cu_3_(HHTP)_2_ SURMOF films are crystalline and highly oriented, with layers parallel to the substrate surface. Most importantly, the one-dimensional pores (i.e., channels) are perpendicular to the substrate surface. In the absence of defects, the channel length corresponds to the film thickness.

The scanning electron microscopy (SEM) images of the samples are shown in Figure S1, revealing that the Cu_3_(HHTP)_2_ SURMOF films exhibit a dense and continuous morphology after both the 20-cycle and 200-cycle samples. Cross-sectional SEM images indicate a film thickness of approximately 70 ± 10 nm for 20 cycles and 540 ± 40 nm for 200 cycles (which are the average values of 4 cross-sectional views of broken samples, and the standard deviation is calculated from them). By increasing the number of synthesis cycles from 20 to 200, the sample thickness increased by approximately 8 times, which is roughly consistent with the expected 10-fold increase in thickness. This essentially linear growth rate (where the film thickness is proportional to the number of synthesis cycles) was also found in previous SURMOF studies.^33,34^ Thus, we use the number of synthesis cycles as a measure for the film thickness, and thus, the channel length.

A three-electrode electrochemical system was constructed to evaluate their electrochemical properties (Figure S2). The Cu_3_(HHTP)_2_ SURMOF film on a gold-coated silicon substrate (0.5 cm^2^) served as the working electrode. A platinum sheet (1 cm^2^) was used as the counter electrode, and an Ag/AgCl electrode was chosen as the reference electrode due to its stability in aqueous systems. To investigate the impact of electrolyte ion types on the capacitor performance and ion transport, a 3 M KCl aqueous solution and pure IL ([BMIM]^+^[TFSI]^−^) were used as electrolytes.

Figures 2a,b and S3 present the CV curves of the Cu_3_(HHTP)_2_ SURMOF electrodes with a KCl electrolyte. It displays nearly rectangular CV curves in the potential range of 0–0.3 V vs Ag/AgCl, with no observable redox peaks. This behavior indicates excellent capacitive performance. The surface areal capacitance calculations further revealed that the double-layer capacitance decreased as the scan rate increased. For example, the surface areal capacitance of the 200-cycle sample dropped from 0.54 F cm^–2^ at a scan rate of 0.1 mV s^–1^ to 0.044 F cm^–2^ at 3000 mV s^–1^ (Figure 2c). This decline in capacitance with increasing scan rate can be attributed to the intrinsic resistance of the active material^35–37^ and/or the relaxation time required for ion diffusion within the electrode structure.^38,39^ While thin samples reach their capacitance limit (or plateau) at low scan rates, thick samples do not achieve this limit even at the lowest studied scan rates. This disparity can be attributed to the increased resistance of ion transport within thicker films, a phenomenon that will be analyzed in greater detail below.

**Figure 2:**
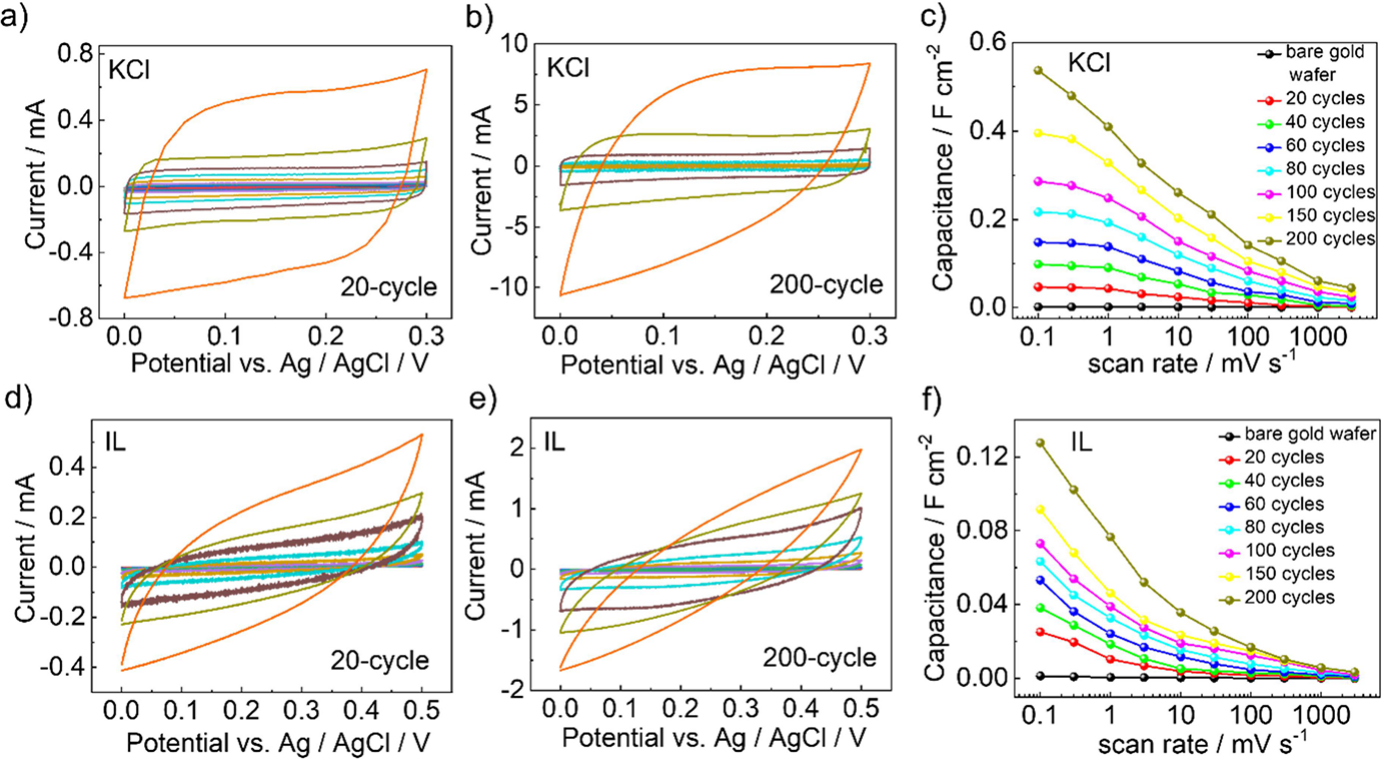
Electrochemical performances of EDLCs with Cu_3_(HHTP)_2_ as the working electrode. CV curves measured at different scan rates for (a) 20- and (b) 200-cycle Cu_3_(HHTP)_2_ samples in the KCl electrolyte, (d) 20- and (e) 200-cycle Cu_3_(HHTP)_2_ samples in pure IL electrolyte. Different colors represent different scan rates, which are 0.1, 0.3, 1, 3, 10, 30, 100, 300, 1000, and 3000 mV s^–1^, from inside to outside. (c,f) Surface areal capacitances of 20- to 200-synthesis-cycle Cu_3_(HHTP)_2_ samples determined from CV versus scan rate with (c) KCl and (f) pure IL as electrolytes.

Next, we replaced the KCl electrolyte with pure IL of the type [BMIM]^+^[TFSI]^−^. The use of IL extends the voltage range without redox reactions at least from 0–0.3 V in KCl to 0–0.5 V. Figures 2d,e and S4 show the CV curves for Cu_3_(HHTP)_2_ electrodes in the IL. The first notable difference between the KCl and IL systems is the CV-curve shape. While KCl produced nearly rectangular curves, indicative of ideal capacitive behavior in EDLCs, the IL system exhibited spindle-shaped curves, reflecting a deviation from the ideal double-layer capacitance but still demonstrating electrochemical activity. As the scan rate increased from 0.1 to 3000 mV s^–1^, the current density increased accordingly, confirming effective double-layer capacitance with IL.

As first finding, we like to state that, for both electrolytes, KCl or pure IL, the scan-rate-dependent capacitance is proportional to the number of synthesis cycles, i.e., the film thickness, see Figures 2c,f and S5 and S10. This proportionality is attributed to the increased specific surface area and pore volume of the 1D channels in the MOF electrodes as their thickness grows. This indicates that the entire material is essentially homogeneous. Since longer pores are more affected by pore-blocking defects (with a homogeneous defect density) than shorter pores, we conclude that defects or other compounds that might block the pores (like binder molecules, which, for instance, are used for pressing the MOF pellets, see Supplementary Table 2 in ref 40) have a minor impact. Thus, it is assumed that the entire pore space is accessible to all ions of the electrolytes.

To quantitatively analyze the capacitance, the dependence of the electrochemical responses on the scan rate was first examined by the analysis following Trasatti and co-workers.^17,25,41^ There, the total capacitance *C*_s_ can be calculated as the sum of the diffusion-controlled (inner) capacitance *C*_i_ and the surface-controlled (outer) capacitance *C*_o_, i.e., *C*_s_ = *C*_i_ + *C*_o_. The total capacitance *C*_s_ can be estimated by extrapolating $1/C(v)=a_{1}\sqrt{v}+C_{s}$ for very small scan rates, i.e., *v* → 0, Figure 3a,c. It is assumed that the dependence of the measured capacitance on the scan rate *v* is based on the diffusion of the electrolytes, resulting in $C(v)=a_{2}/\sqrt{v}+C_{o}$ (*a*_1_ and *a*_2_ are free constants.) Thus, extrapolating for infinite fast scan rates (i.e., $1/\sqrt{v}\to 0$ ) allows an estimation of the outer capacitance *C*_o_, Figure 3b,d. The estimation of *C*_s_ and *C*_0_ of 20- and 200-cycle samples in KCl and IL electrolytes is shown in Figure 3a–d, and the plots of the other samples are shown in Figures S12 and S13. The determined total capacitance *C*_s_ and outer capacitance *C*_0_ for the samples of different thicknesses are shown in Figure 3e,f. For KCl, it shows that both the total and outer capacitance increase essentially linearly with the film thickness (i.e., the number of synthesis cycles). A detailed inspection shows that the linear growth slightly attenuates for the sample with more than 150 synthesis cycles. Moreover, it shows that the outer capacitance is only slightly smaller than the total capacitance; this means that the entire material is accessible by the electrolyte. Different results are found for IL as the electrolyte: The total capacity also increases with the film thickness, but the growth rate is less than linear. Also, the values of the total capacitance for IL are approximately 1.5–3.5 times smaller than those for KCl. The difference increases with an increasing film thickness. In addition, for the IL, the outer capacitance is significantly smaller than the total capacitance. The ratio between them is roughly 4. This means that a major part of the MOFs is not accessible to the IL electrolyte at fast scan rates.

**Figure 3:**
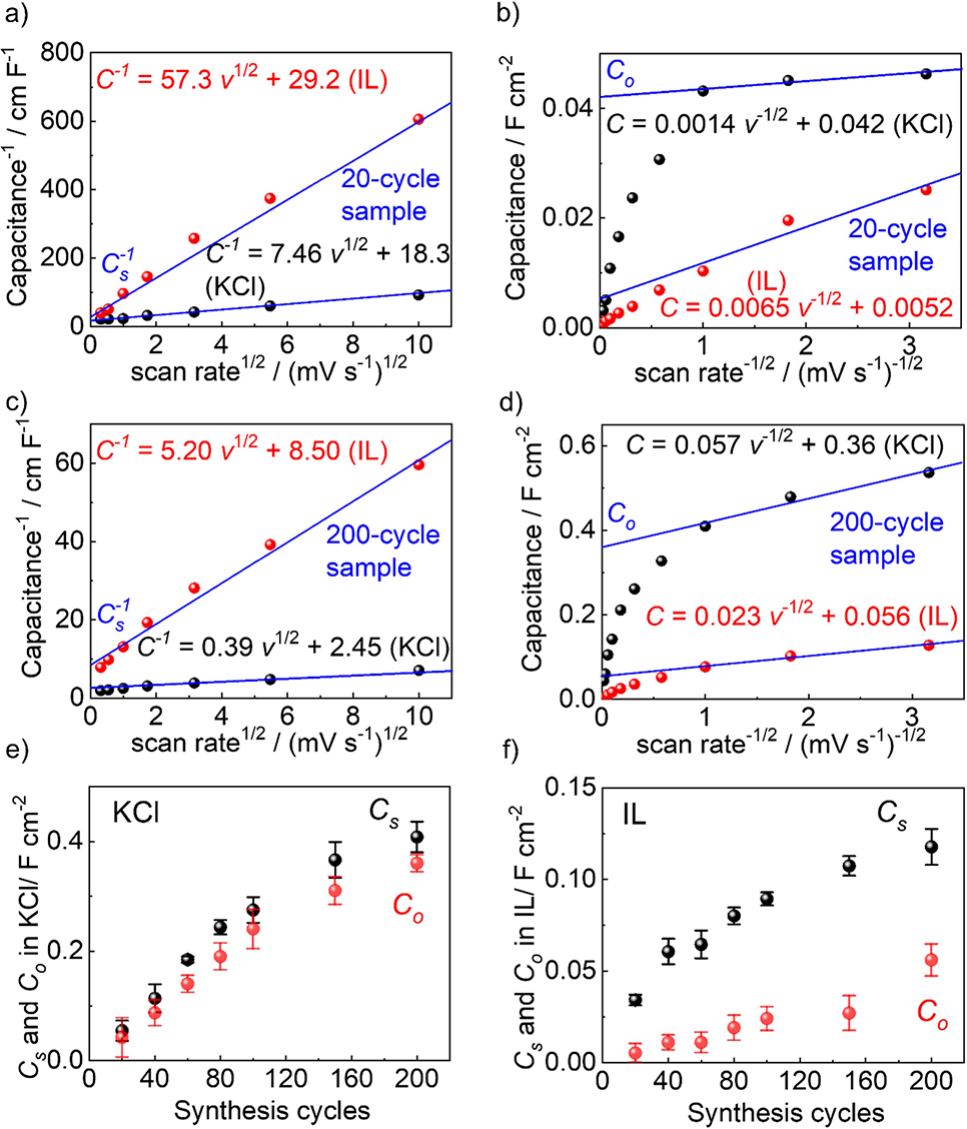
(a,c) Reciprocal of capacitance *C* ^–1^ vs square root of scan rate (ν^1/2^), with extrapolation to ν = 0 to estimate the total surface capacitance *C*_s_ of (a) 20-cycle and (c) 200-cycle sample. The data of the IL are shown in red and KCl in black in all panels (a–d). (b,d) Surface areal capacitance vs reciprocal square root of scan rate (ν^–1/2^), with extrapolation to ν → ∞ to estimate the outer surface capacitance *C*_o_ of (b) 20-cycle and (d) 200-cycle sample. (e,f) Total capacitance *C*_s_ (black) and the outer capacitance *C*_o_ (red) vs synthesis cycles for (e) KCl and (f) IL as electrolytes.

Based on the specific surface area of 554 m^2^ g^–1^ (ref 22), the density of 1.05 g cm^–3^ (determined from the perfect MOF structure), the film thicknesses determined by SEM, and the total capacitance of 0.055, 0.41, 0.034, and 0.12 F cm^–2^, the determined (total) capacitances normalized to the MOF surface area are 1340 and 1300 μF cm^–2^ for the 20- and 200-cycle samples for KCl and 841 and 375 μF cm^–2^ for 20- and 200-cycle samples for the IL, respectively. This means that while the capacitance with respect to the MOF surface area is constant for KCl, the capacitance decreases strongly for the IL. Apart from kinetic effects, which make the EDLC less accessible for the IL, the smaller capacitance for the IL is also attributed to the larger ionic sizes of [BMIM]^+^ and [TFSI]^−^ compared to K^+^ and Cl^–^, reducing charge density at the electrode–electrolyte interface.

Additionally, the method introduced by Dunn and co-workers^26,41^ was employed to analyze the contributions of the diffusive and capacitive current for samples synthesized with 20 and 200 cycles (Figure 4). The method is based on the current which is attributed to surface-controlled processes (*i*_capacitive_) and diffusion-controlled processes (*i*_diffusion_), i.e., *i*_total_ = *i*_capacitive_ + *i*_diffusion_. While *i*_diffusion_ should be proportional to $\sqrt{v}$ , *i*_capacitive_ should be proportional to the scan rate *v*. Thus, the current can be written as

$$i_{\mathrm{total}}/\sqrt{v}=a_{1}\sqrt{v}+a_{2}$$ 1

where $a_{1}\sqrt{v}$ describes the surface-controlled (nondiffusion-controlled) part and *a*_2_ describes the diffusion-controlled part. As shown in Figures 4 and S15, at low scan rates of 1 mV s^–1^, the diffusion-controlled capacitance dominates the overall capacitance performance, while at higher scan rates, the nondiffusion-controlled capacitance becomes more significant. Generally, the part of the nondiffusion-controlled capacitance is larger for KCl than for IL.

**Figure 4:**
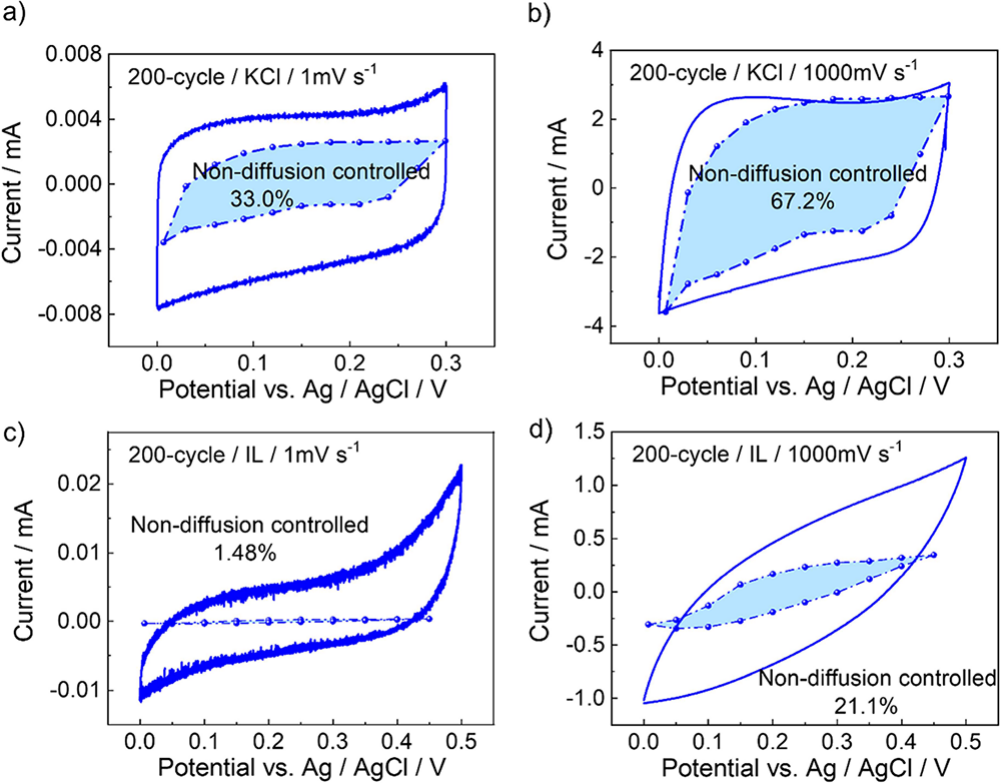
CV curves with marked nondiffusion-controlled contributions in the total capacitance calculated by the method introduced by Dunn et al.^26,41^ The nondiffusion-controlled part (light-blue area) corresponds to $a_{1}\sqrt{v}$ in eq 1, and the diffusion-controlled part (which is the rest) corresponds to *a*_2_ in eq 1. The percentage values are the ratios of the nondiffusion-controlled areas (i.e., capacitance) versus the total area. The scan rates are (a) 1 mV s^–1^ and (b) 1000 mV s^–1^ for KCl and (c) 1 mV s^–1^ and (d) 1000 mV s^–1^ for the IL, see labels. The same sample made by 200 synthesis cycles was used.

To further analyze the ion transport within the Cu_3_(HHTP)_2_ SURMOF electrodes, we studied the scan rate dependence of CV currents using the power law equation *i* = *av* ^*b*^. (In this equation, current *i* is proportional to scan rate *v* to power of *b*.) Here, *b* serves as a key parameter: *b* = 0.5 indicates a diffusion-limited regime, as characterized by the Randles–Ševčík equation,^28^ while *b* = 1 indicates surface-controlled processes. For this analysis, we systematically fit the peak currents vs scan rates for both KCl and IL. Peak currents at 0 and 0.3 V were selected for KCl (Figures 5a and S16), while those at 0 and 0.5 V were chosen for IL (Figures 5b and S17). Logarithmic plots of peak currents vs scan rates allowed us to extract *b*-values from the slope of linear fits, providing insights into the diffusion and charge-transfer mechanisms for each electrolyte.

**Figure 5:**
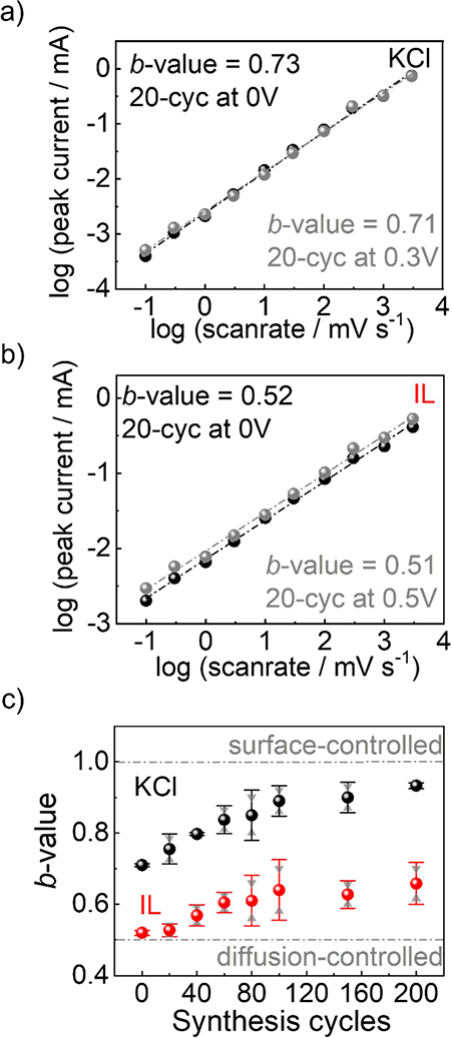
*b*-value determined from the peak currents at scan rates from 0.1 to 3000 mV s^–1^ for the 20-cycle sample in (a) KCl and (b) IL electrolytes. (More data are shown in the SI.) (c) *b*-value vs synthesis cycles. The black spheres are the KCl data, and the IL is in red. The average data with standard deviations as error bars are shown. The individual data points (from panels a and b as well as Figures S16 and S17) are shown as gray triangles.

Figure 5c illustrates the relationship between the determined *b*-values and the number of synthesis cycles for KCl and IL as electrolytes. The *b*-values show an increasing trend for both electrolytes with an increasing film thickness. In particular, for the samples with 20–100 synthesis cycles, a clear (approximately linear) increase of *b* with increasing cycle number can be seen. When comparing the two systems, the *b*-value for KCl (almost approaching 1) is significantly higher than that for the IL. In agreement with the analyses above, this suggests that, for KCl, the diffusion of the ions is less limiting to the kinetics, and the current is more influenced by the resistance at the electrode–electrolyte interface, i.e., surface-limited.^27^ (The surface-controlled range involves the formation of ion layers at the surface, here within the MOF pores. Surface control also encompasses electron transfer within the MOF. However, the high conductivity of the MOF suggests that its electronic resistance is likely not the limiting factor.) In contrast, the *b*-value for the IL system is closer to 0.5, indicating that the current is primarily limited by the diffusion of the ions.^42^ This difference is attributed to a larger ion size and higher viscosity of the IL compared to aqueous KCl solution,^16^ causing a larger diffusion transport resistance. This contributes to the lower *b*-value.^43^

As the thickness of the film increases, the *b*-values increase from 0.71 to 0.93 in KCl and from 0.52 to 0.66 for the pure IL, Figure 5c. In EDLCs, various factors can affect ion transport. For example, MOF electrodes with large pore sizes can induce stronger screening electrostatic interactions due to increased image charges on the pore walls, thereby hindering the ion mobility within the pores.^14^ Although a higher specific surface area in MOFs can enhance the capacitance, it also presents challenges for efficient ion transport into and out of the active layers.^44,45^

When pure IL is used as the electrolyte, its larger ion size and higher viscosity make ion transport significantly more difficult in both the bulk electrolyte and the MOF pores compared to KCl. As a reference, the diffusion coefficient of 3 M KCl was determined to be approximately 2 × 10^–9^ m^2^ s^–1^ at room temperature.^46^ For [BMIM]^+^[TFSI]^−^, it was determined to be 1.74 and 1.03 × 10^–11^ m^2^ s^–1^ for the cation and the anion, respectively.^47^ As a result, charge transport in capacitors using pure IL is predominantly diffusion-controlled, whereas in KCl, it is surface-controlled. As the thickness of the MOF electrode increases, both systems show an increase in *b*-values, indicating a growing contribution from surface-controlled processes. This can be attributed to the larger specific surface area and pore volume provided by thicker films, allowing more ions to be stored, thereby enhancing capacitance. An increased film thickness also hinders ion transport within the MOF pores, adding resistance to the formation of ion layers on the MOF pore surfaces. This shift further emphasizes the role of surface-controlled processes as rate-limiting factors, as reflected in the increased *b*-values. A sketch that illustrates the transport of electrolyte ions in the bulk electrolyte and within the MOF electrode pores is shown in Figure S18. More features and details of the ion transport need to be explored, especially the correlation between the capacity changes and the size of the electrolyte ions.

The study shows that the task of finding the ideal channel length depends on various factors. For a highly mobile electrolyte (like KCl), thick films with long pore channels enable large capacitances with respect to the substrate area, while the surface capacitance with respect to the specific MOF surface area remains constant. This allows the realization of high-power supercapacitors. On the other hand, for a less mobile electrolyte (like the studied IL, which possesses advantages like a wider voltage range and advantages in terms of safety concerns), very short channel lengths are important for high efficiency. There, unlike in long channels, the transport resistance of the ions is small and does not limit the supercapacitor performance.

## Conclusions

In summary, we synthesized 2D-cMOF Cu_3_(HHTP)_2_ films with varying thicknesses and evaluated their performance as working electrodes in EDLCs. By using two types of electrolytes, aqueous KCl and pure IL [BMIM]^+^[TFSI]^−^, we systematically investigated the impact of the channel length of the pores on the EDLC capacitance and the ion transport kinetics within the MOF electrodes. The CV data are analyzed by various methods, including those introduced by Trasatti and Dunn. The determined *b*-value from the Randles–Sevcik equation increases with film thickness, indicating a transition from diffusion-controlled to surface-controlled current behavior, highlighting the growing influence of surface processes in thicker films. The analysis of the total capacitance shows that, while the entire pore space seems to be accessible by the ions of the electrolyte, the film thickness (i.e., the channel length) can have a dramatic impact on the capacitance, depending on the electrolyte. For highly mobile aqueous KCl solution, the capacitance per specific MOF surface area is not affected by the film thickness; thus, thicker films result in larger capacitances normalized to the substrate area. On the other hand, using IL as the electrolyte shows a strong diffusion transport limitation, which results in decreasing performance with increasing channel length. This study provides valuable insights into the design and optimization of electrodes made of 2D c-MOF and also other materials for energy storage applications. In future studies, the effect of the pore structure and functionality along with electrolyte optimization needs to be explored in combination with the effect of the channel length. This will allow us to develop the ideal material for high-performance supercapacitors and other energy storage technologies.

## Supplementary Material



## Data Availability

The raw data are available on Zenodo (https://doi.org/10.5281/zenodo.14961573).

## Notes

Additional experiment methods, SEM, CV, and *b*-value fitting data (PDF)
