# Supplementary material for: Impact of the Channel Length in Nanoporous Electric Double-Layer Capacitors on the Charge Transport Explored by Metal–Organic Framework Films
Source: ACS Phys Chem Au. 2025 Mar 4;5(3):266–73. doi: 10.1021/acsphyschemau.4c00104 (PMC12123543; doi:10.1021/acsphyschemau.4c00104)
Supplement: Supplementary file 1 [file pg4c00104_si_001.pdf]

Supporting Information:

Impact of the Channel Length in Nanoporous Electric Double Layer Capacitors on the Charge Transport Explored by Metal-Organic-Framework Films

Yidong Liu, [a] *Abhinav Chandresh*[b] *and Lars Heinke*[a,b],*

[a] Institute of Functional Interfaces (IFG), Karlsruhe Institute of Technology (KIT), Hermann-von-Helmholtz-Platz 1, 76344 Eggenstein-Leopoldshafen, Germany.

[b] Physical Chemistry, Institute of Chemistry and Biochemistry, Freie Universität Berlin, Arnimallee 22, 14195 Berlin, Germany. E-mail: Lars.Heinke@FU-Berlin.de

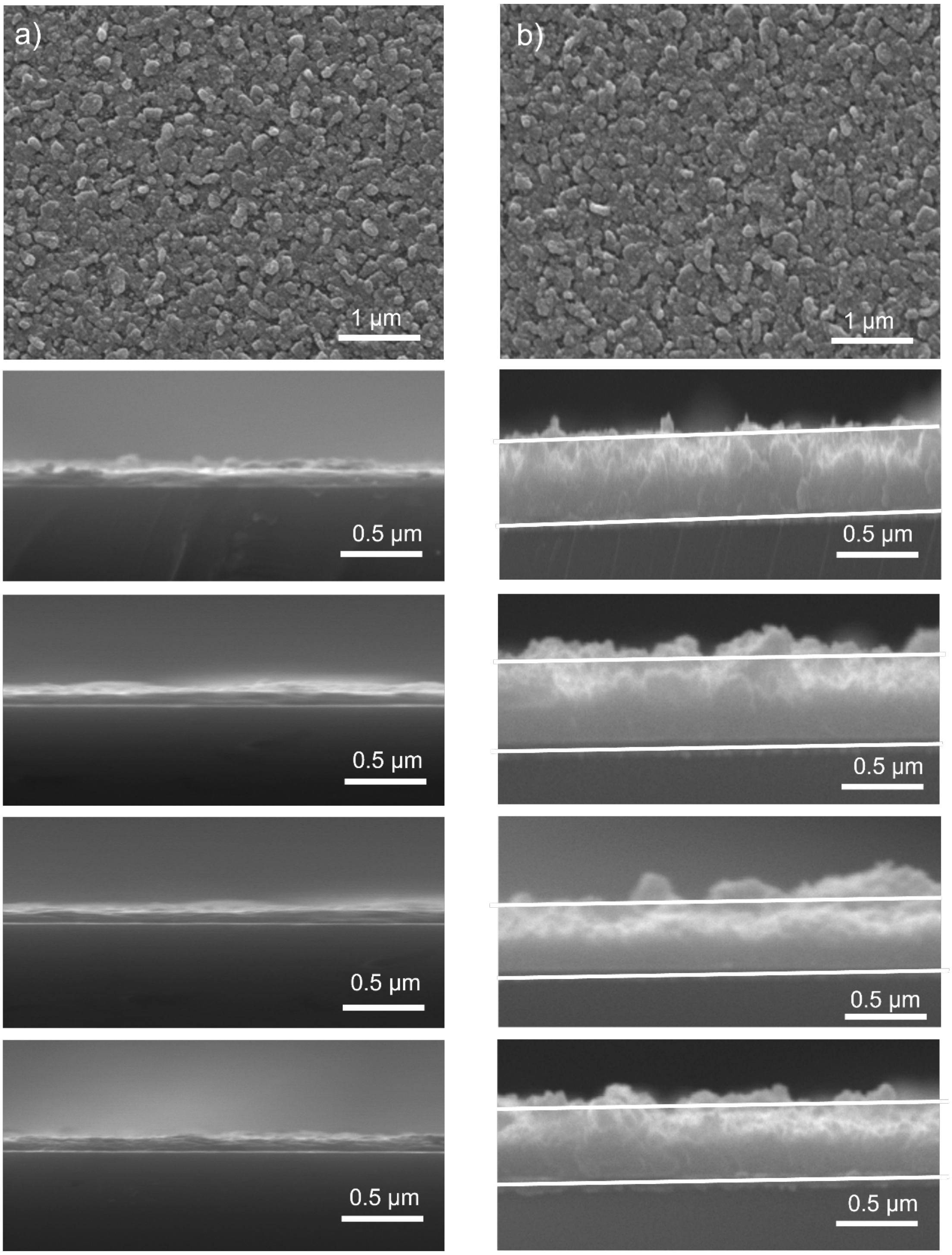

Figure S1. SEM images of the a) 20-cycle and b) 200-cycle $Cu_3(HHTP)_2$ SURMOF thin films. Top view (above) and 4 cross-section views of broken samples (below) are for thickness calculation. The thickness for 20-cycles samples are 85 nm, 65 nm, 55 nm and 70 nm, the average thickness is approximately 70 ± 10 nm. The thickness for 200-cycles samples are 555 nm, 590 nm, 495 nm and 515 nm, the average thickness is approximately 540 ± 40 nm.

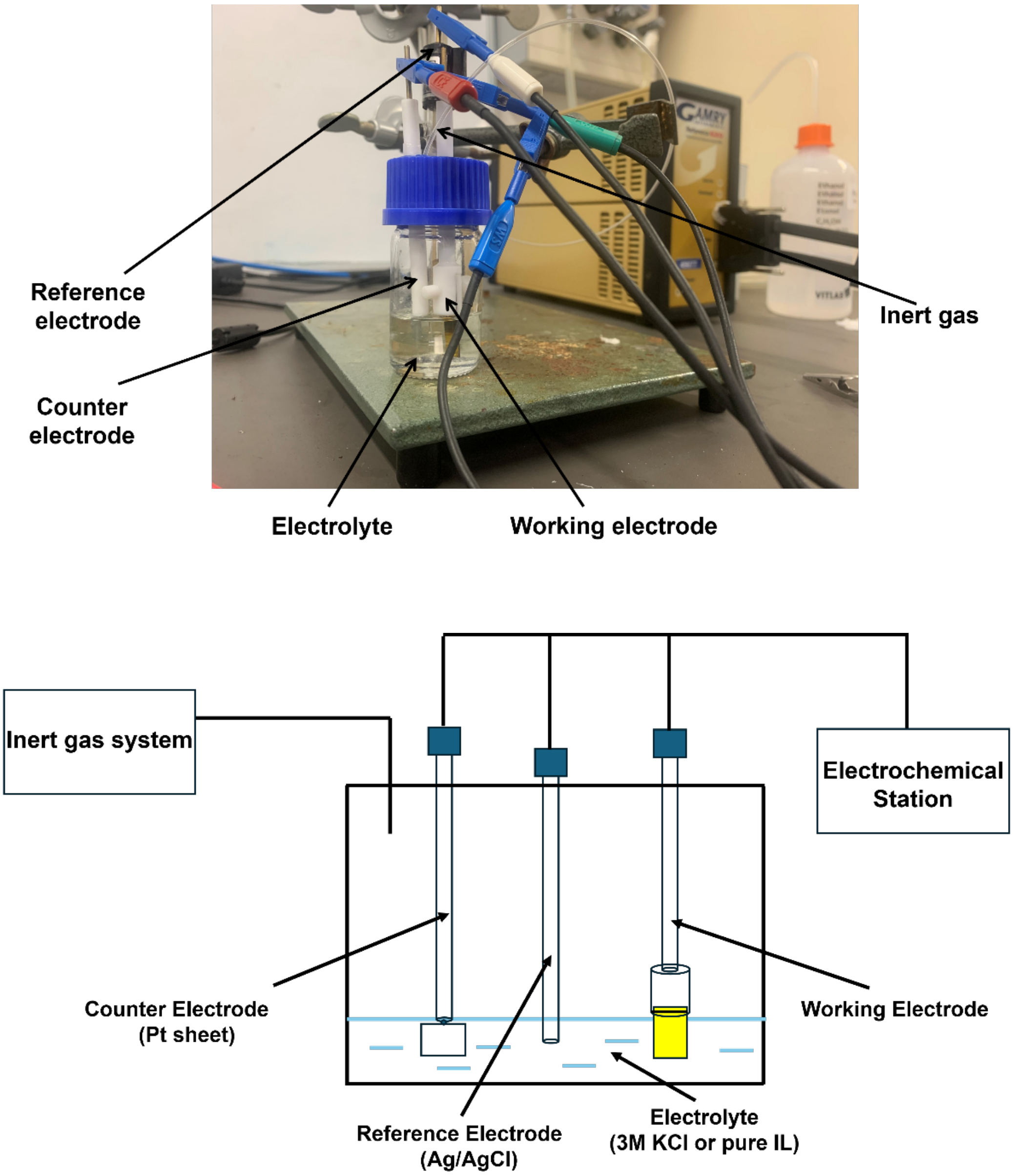

Figure S2. Picture (top) and sketch (down) of the three-electrode system.

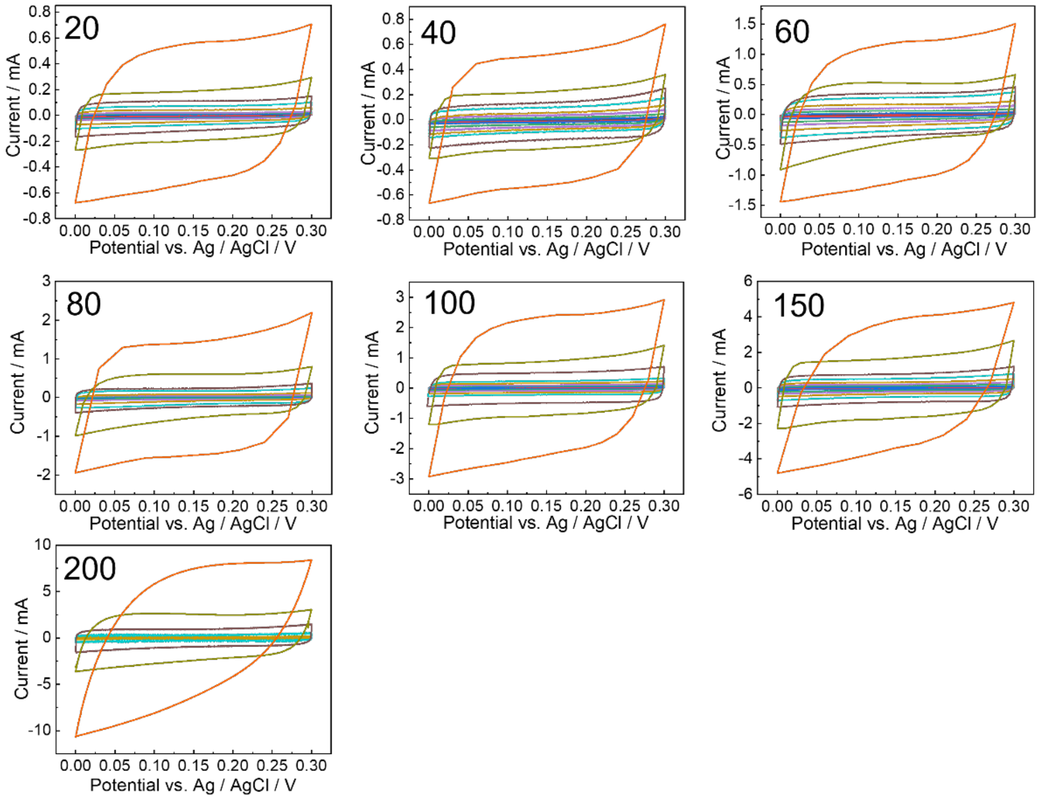

Figure S3. CV measurements of different thickness $Cu_3(HHTP)_2$ samples (batch 1) in three-electrode system with KCl as electrolyte. For every pattern, different colors represent different scan rates, from inside to outside they are 0.1, 0.3, 1, 3, 10, 30, 100, 300, 1000, 3000 mV s^{-1}. The number in the upper left corner represents the number of cycles in the synthesis process of the sample.

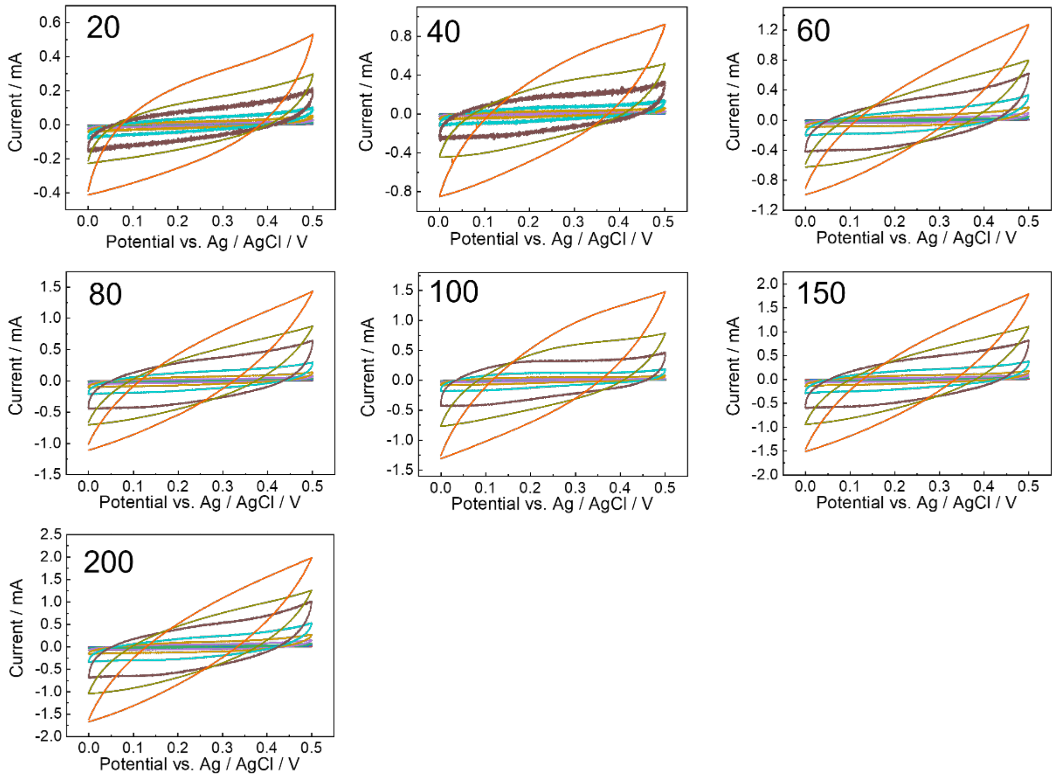

Figure S4. CV measurements of different thickness $Cu_3(HHTP)_2$ samples (batch 1) in three-electrode system with pure IL $[BMIM]^+[TFSI]^-$ as electrolyte. For every pattern, different colors represent different scan rate, from inside to outside they are 0.1, 0.3, 1, 3, 10, 30, 100, 300, 1000, 3000 mV s^{-1}. The number in the upper left corner represents the number of cycles in the synthesis process of the sample.

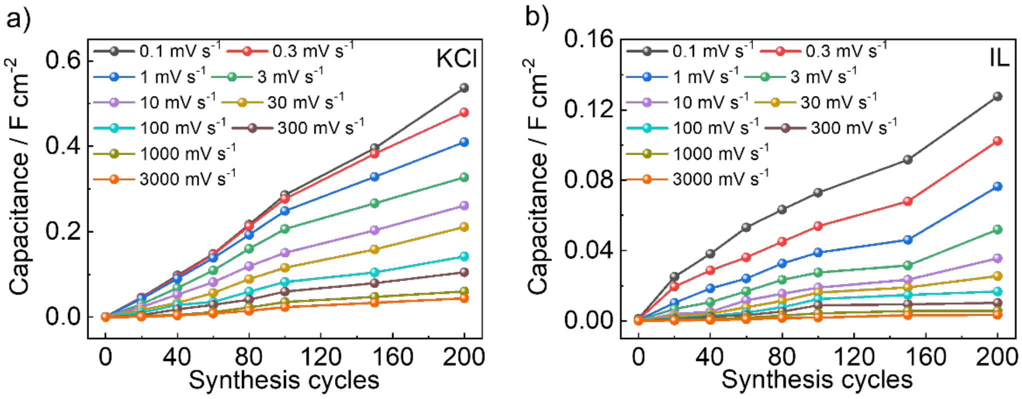

Figure S5. Surface areal capacitance *vs.* synthesis cycles for $Cu_3(HHTP)_2$ MOF electrode with different scan rates from 0.1 to 3000 mV s^{-1} in **a)** KCl and **b)** pure IL.

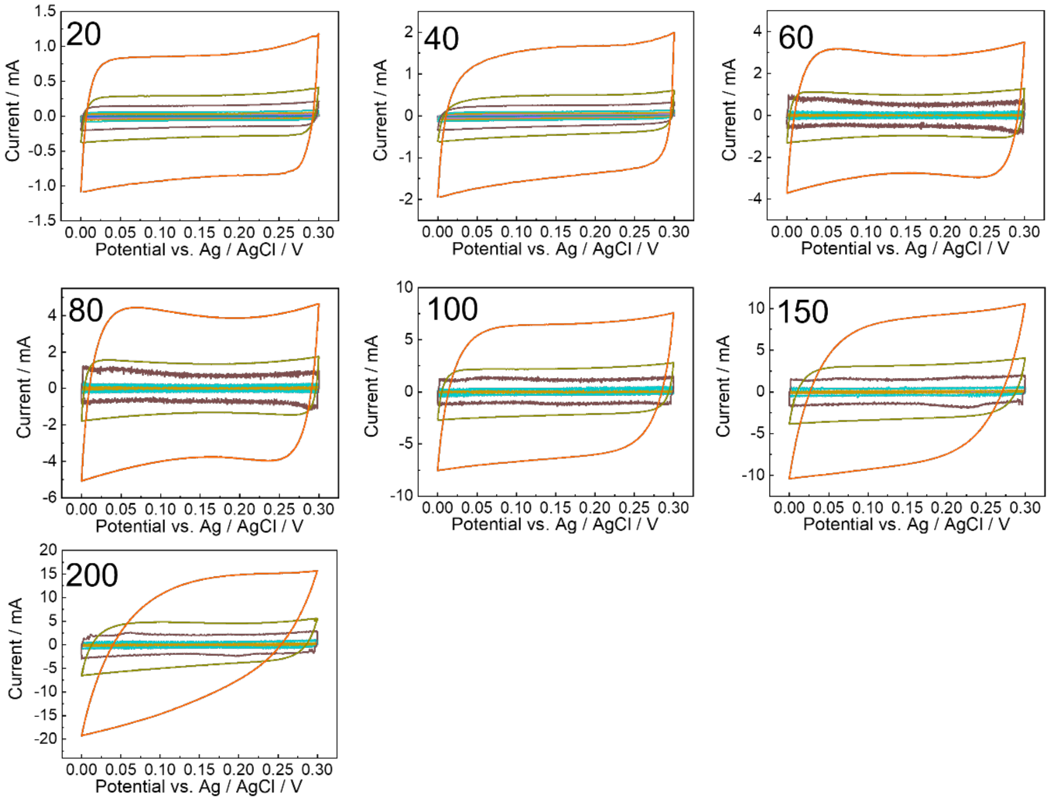

Figure S6. CV measurements of different thickness $Cu_3(HHTP)_2$ samples (batch 2) in three-electrode system with 3M KCl as electrolyte. For every pattern, different colors represent different scan rates, from inside to outside they are 0.1, 0.3, 1, 3, 10, 30, 100, 300, 1000, 3000 mV s^{-1}. The number in the upper left corner represents the number of cycles in the synthesis process of the sample.

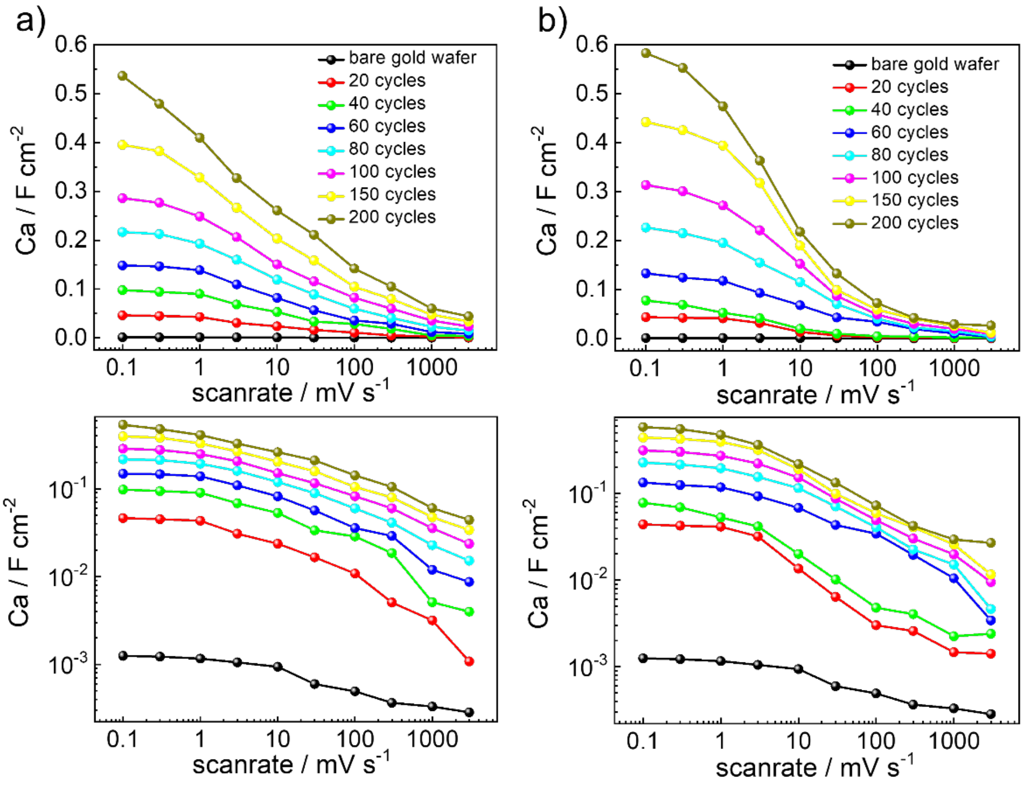

Figure S7. Specific capacitance *vs.* scan rate for a) batch 1 and b) batch 2 $Cu_3(HHTP)_2$ samples with different synthesis cycle numbers from 20 to 200 in 3M KCl solution. The plots below are derived from the above plots with the y-axis converted to a logarithmic scale. In batch 1, the specific capacitance of the 20-cycle sample dropped from 4.6×10^{-2} F cm^{-2} at a scan rate of 0.1 mV s^{-1} to 1.1×10^{-3} F cm^{-2} at 3000 mV s^{-1}. A similar trend was observed for the 20-cycle sample in batch 2, where the specific capacitance decreased from 4.4×10^{-2} F cm^{-2} to 1.4×10^{-2} F cm^{-2} over the same range of scan rates.

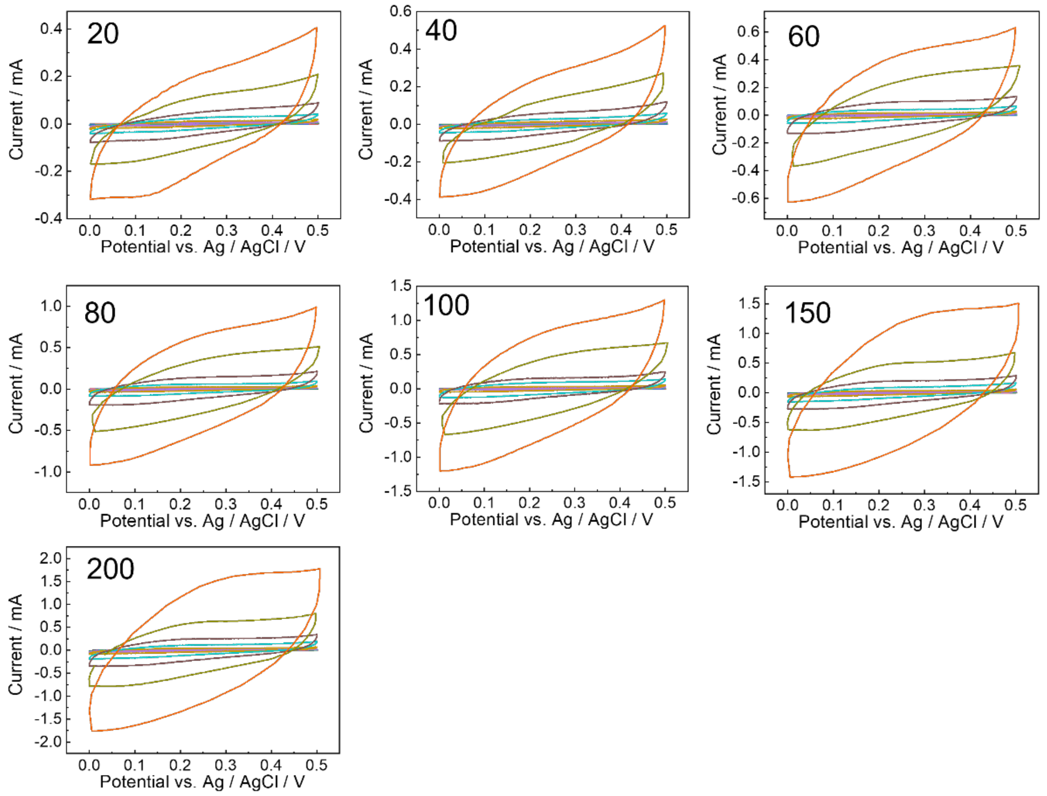

Figure S8. CV measurements of different thickness $Cu_3(HHTP)_2$ samples (batch 2) in three-electrode system with pure IL $[BMIM]^+[TFSI]^-$ as electrolyte. For every pattern, different colors represent different scan rate, from inside to outside they are 0.1, 0.3, 1, 3, 10, 30, 100, 300, 1000, 3000 mV s^{-1}. The number in the upper left corner represents the number of cycles in the synthesis process of the sample.

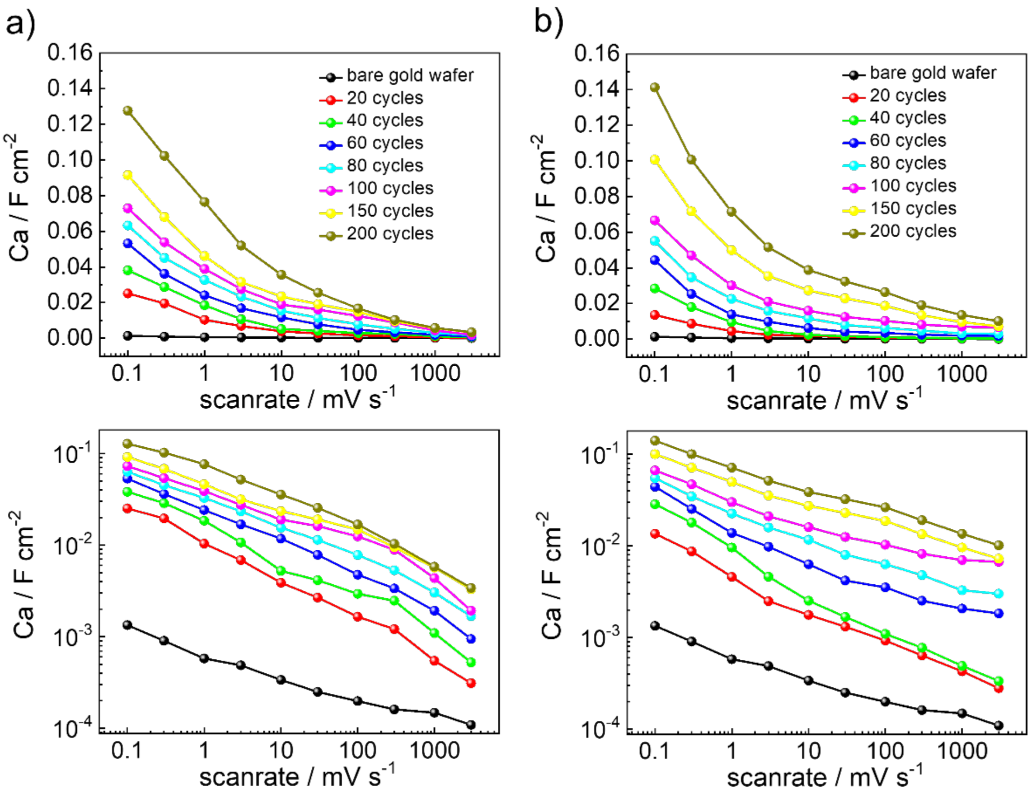

Figure S9. Specific capacitance *vs.* scan rate for a) batch 1 and b) batch 2 $Cu_3(HHTP)_2$ samples with different synthesis cycle numbers from 20 to 200 in pure IL $[BMIM]^+[TFSI]^-$. The plots below are derived from the above plots with the y-axis converted to a logarithmic scale.

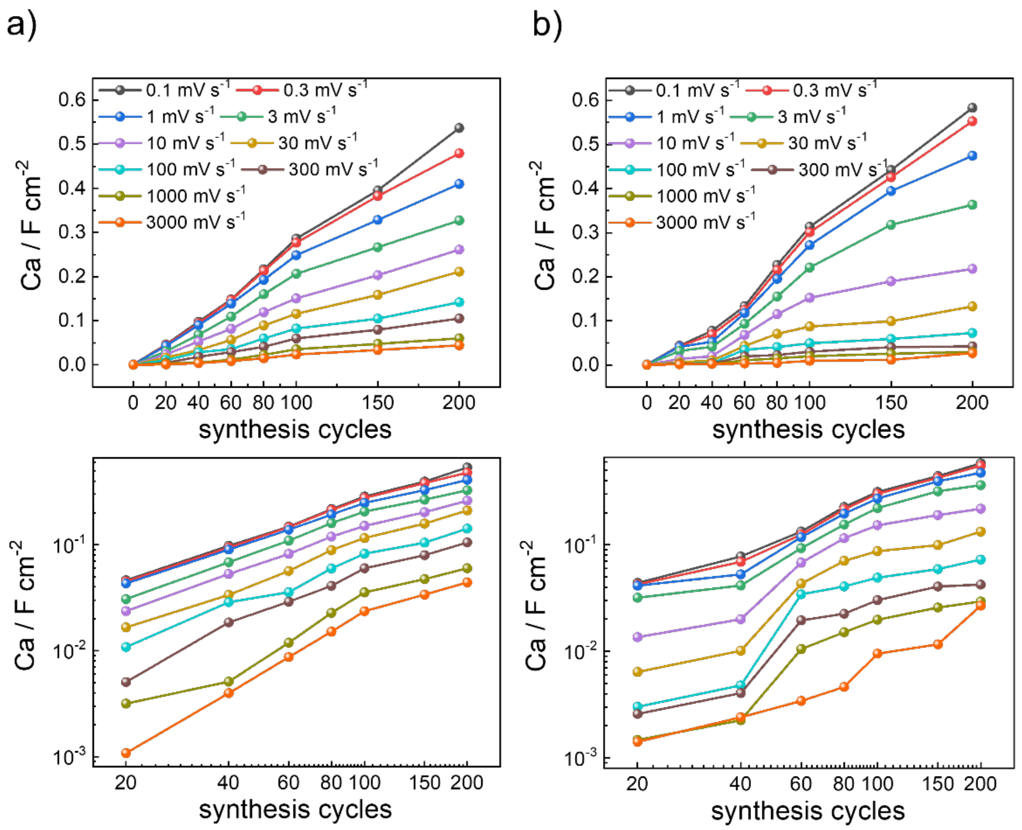

Figure S10. Specific capacitance *vs.* synthesis cycles for a) batch 1 and b) batch 2 $Cu_3(HHTP)_2$ samples with different scan rates from 0.1 to 3000 mV s^{-1} in KCl solution. The plots below are derived from the above plots with the x- and y-axis converted to a logarithmic scale. As the sample thickness increased, the maximum specific capacitance also rose, from 4.6×10^{-2} F cm^{-2} for the 20-cycle sample to 5.4×10^{-1} F cm^{-2} for the 200-cycle sample in batch 1 and the number for batch 2 sample is 4.4×10^{-2} F cm^{-2} to 5.8×10^{-1} F cm^{-2}.

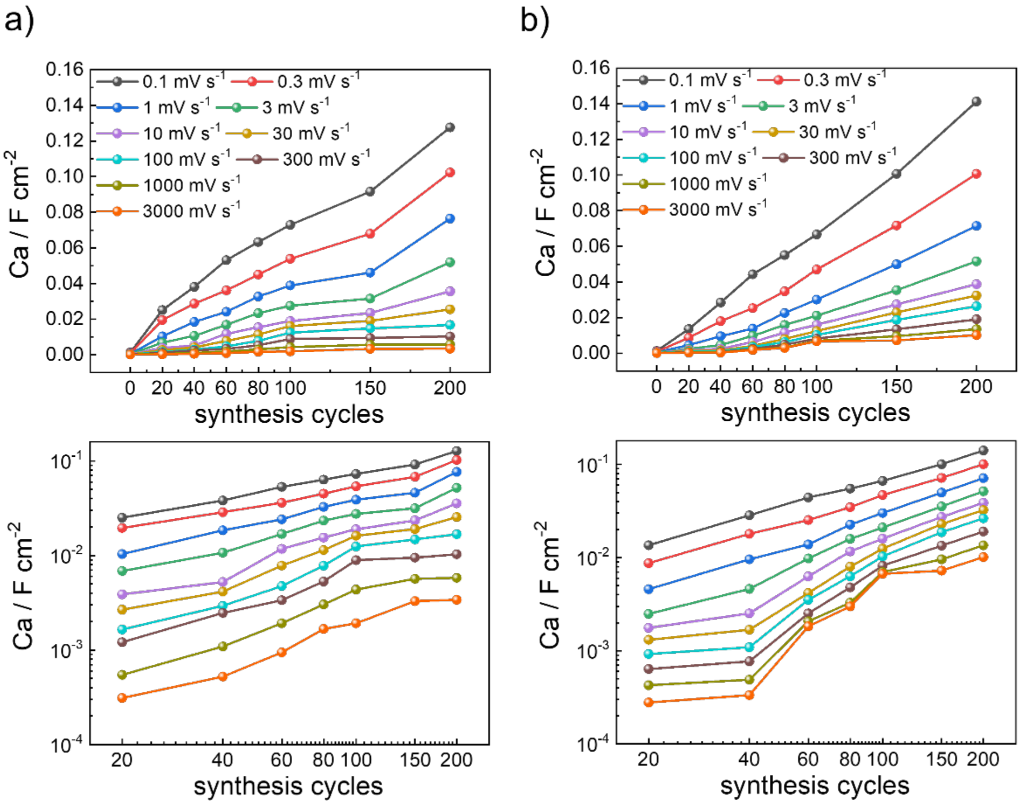

Figure S11. Specific capacitance *vs.* synthesis cycles for a) batch 1 and b) batch 2 $Cu_3(HHTP)_2$ samples with different scan rates from 0.1 to 3000 mV s^{-1} in pure IL. The plots below are derived from the above plots with the x- and y-axis converted to a logarithmic scale. In batch 1, the maximum capacitance increased from 2.5×10^{-2} F cm^{-2} for the 20-cycle sample to 1.3×10^{-1} F cm^{-2} for the 200-cycle sample, while for batch 2, it increased from 1.3×10^{-2} F cm^{-2} to 1.4×10^{-1} F cm^{-2}.

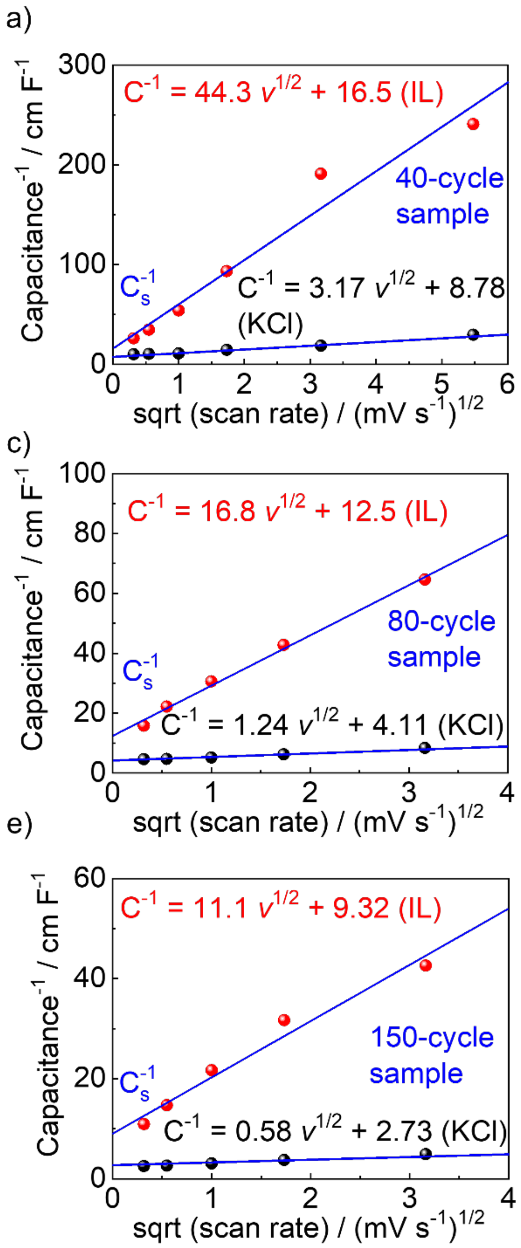

Figure S12. Reciprocal of surface areal capacitance (C^{-1}) *vs.* square root of scan rate ($\nu^{1/2}$), with extrapolation to $\nu = 0$ to estimate the total surface capacitance (C_s) of 40- to 150-cycle samples.

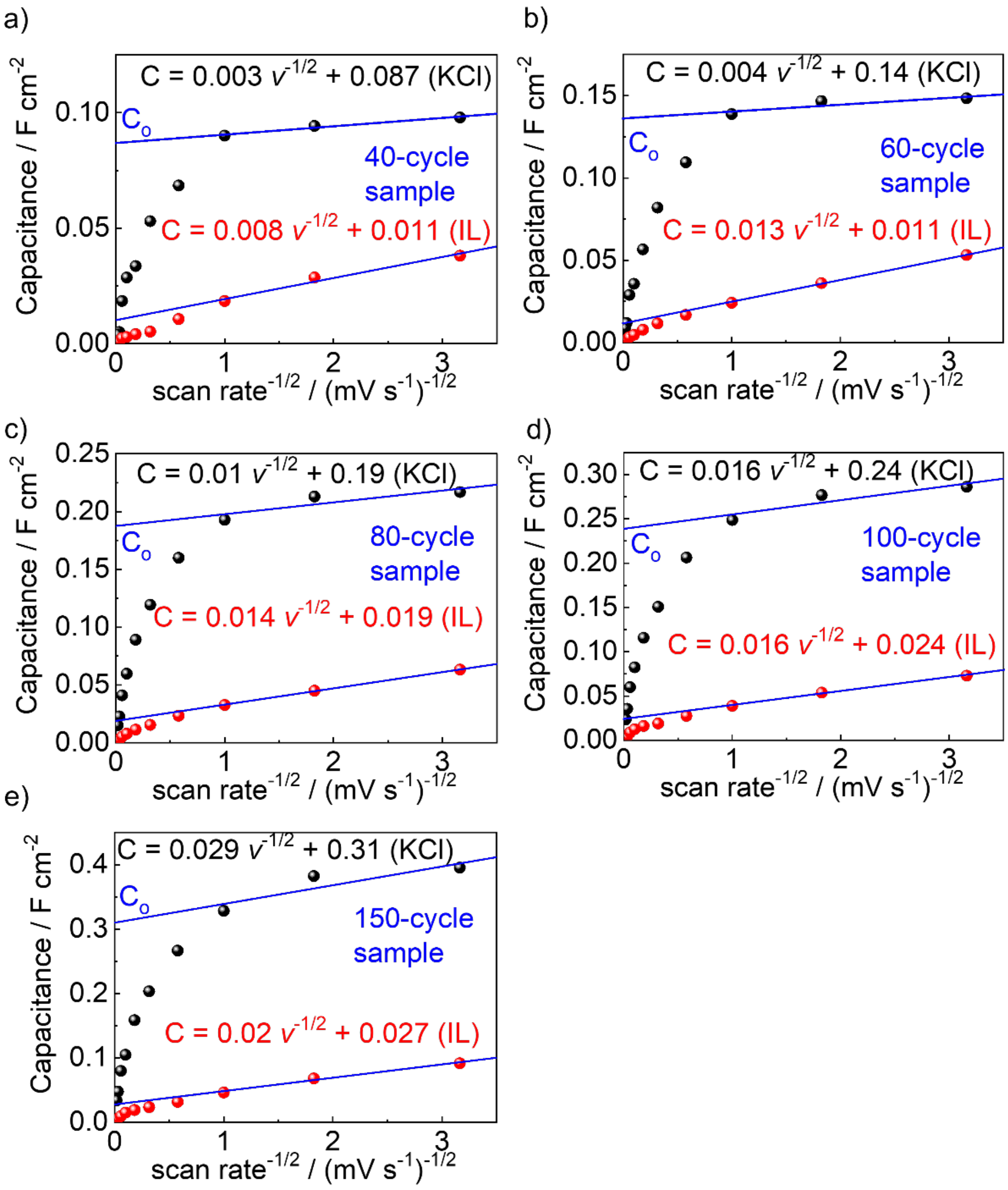

Figure S13. Surface areal capacitance (*C*) *vs.* reciprocal of the square root of scan rate ($\nu^{-1/2}$), with extrapolation to $\nu \rightarrow \infty$ to estimate the outer surface capacitance (C_o) of 40- to 150-cycle samples.

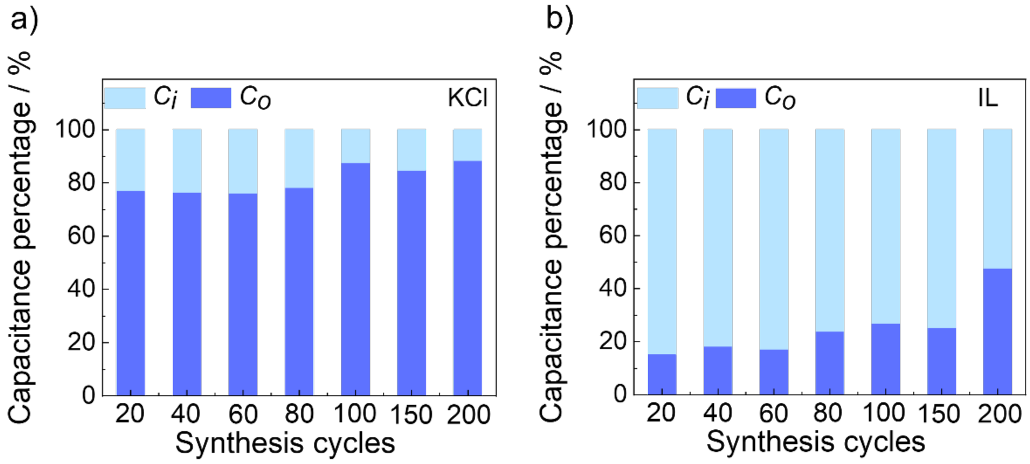

Figure S14. Trasatti analysis of samples with different synthesis cycles in **a)** KCl and **b)** IL.

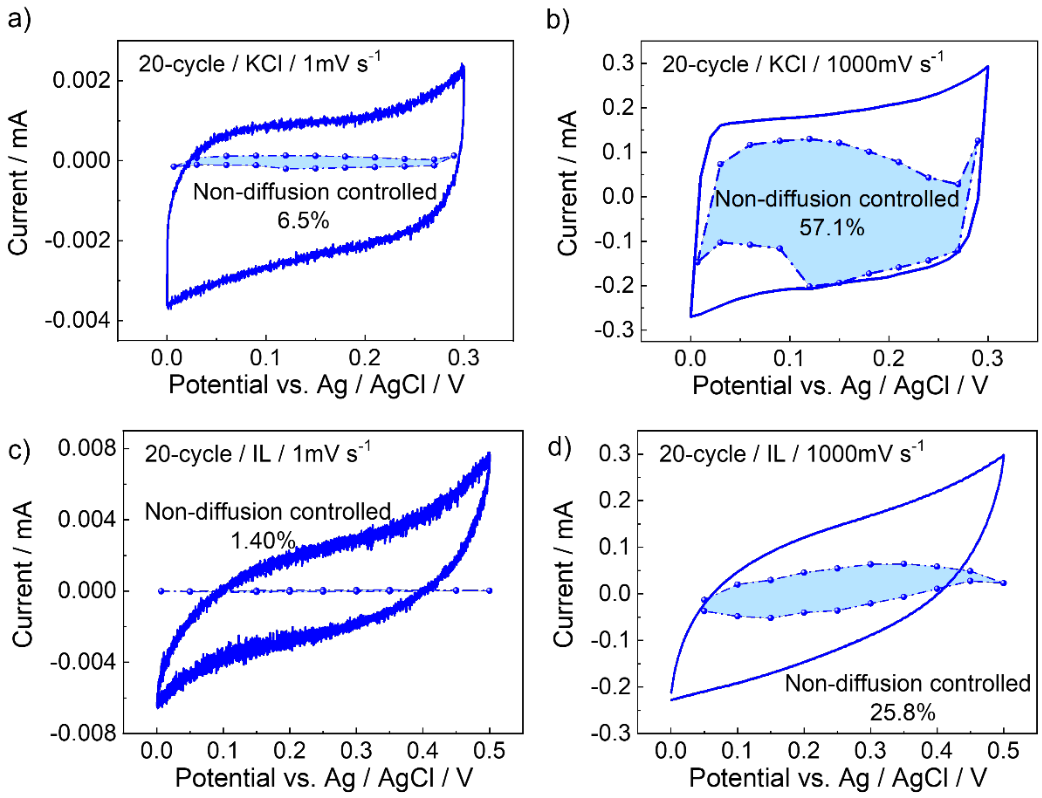

Figure S15. Dunn analysis of 20-cycle sample in KCl and IL electrolyte with scan rate of 1 mV s^{-1} and 1000 mV s^{-1}.

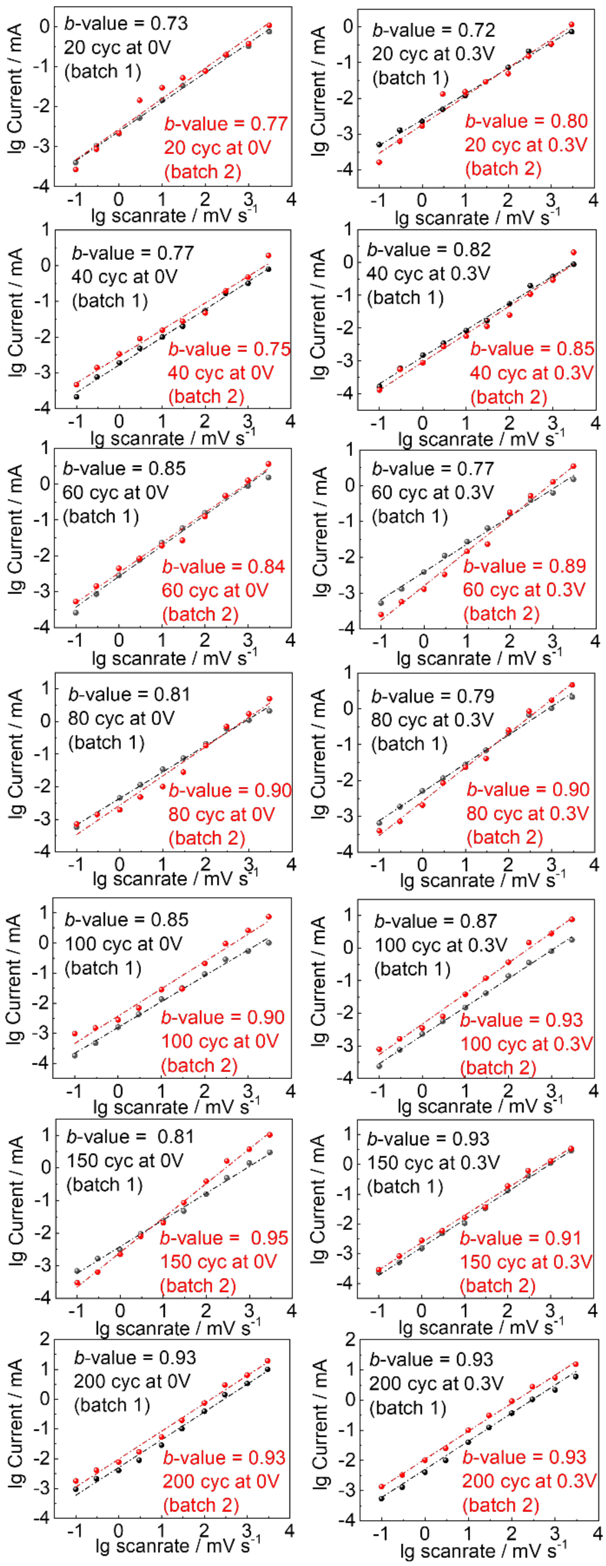

Figure S16. The *b*-values according to $i = av^b$, fitted to CV data collected at scan rates between 0.1 and 3000 mV s^{-1} for batch 1 (black) and batch 2 (red) samples. Saturated KCl is the electrolyte. In the figure, the *b*-value, synthesis cycles and the sampling point location are marked.

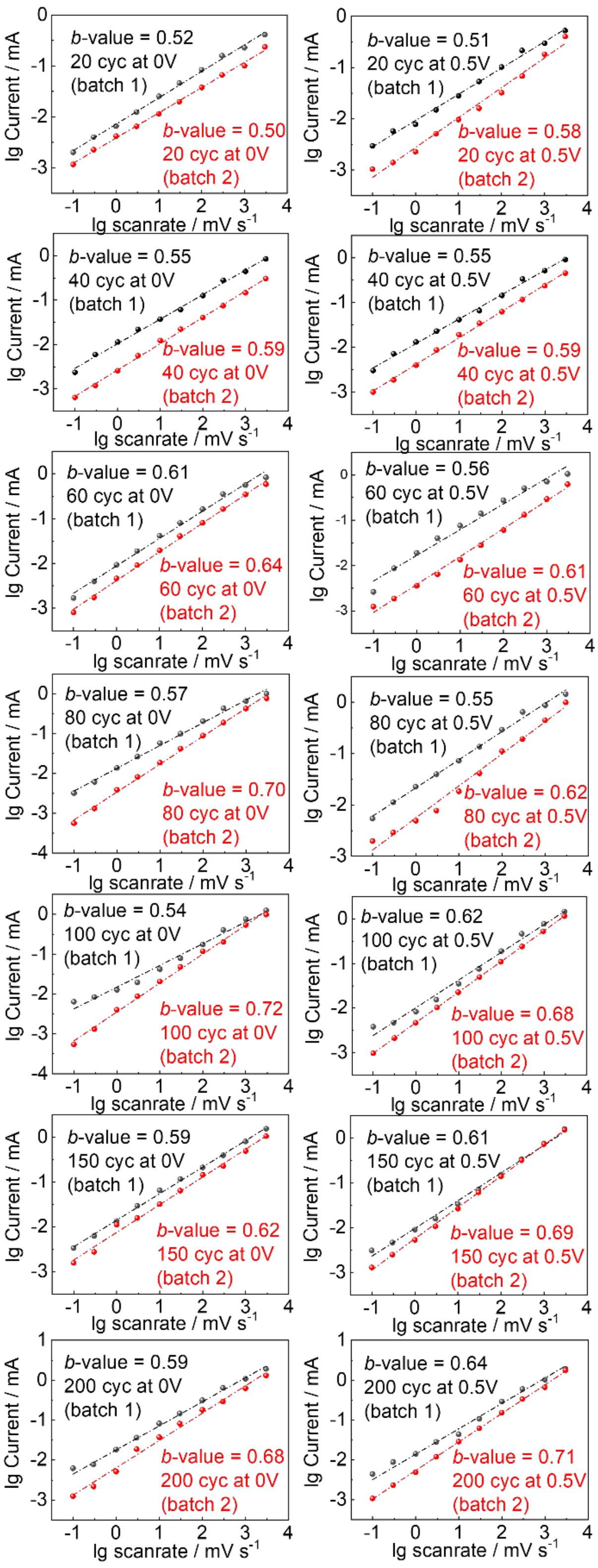

Figure S17. The *b*-values according to $i = av^b$, fitted to CV data collected at scan rates between 0.1 and 3000 mV s^{-1} for batch 1 (black) and batch 2 (red) samples. Pure IL $[BMIM]^+[TFSI]^-$ is the electrolyte. In the figure, the *b*-value, synthesis cycles and the sampling point location are marked.

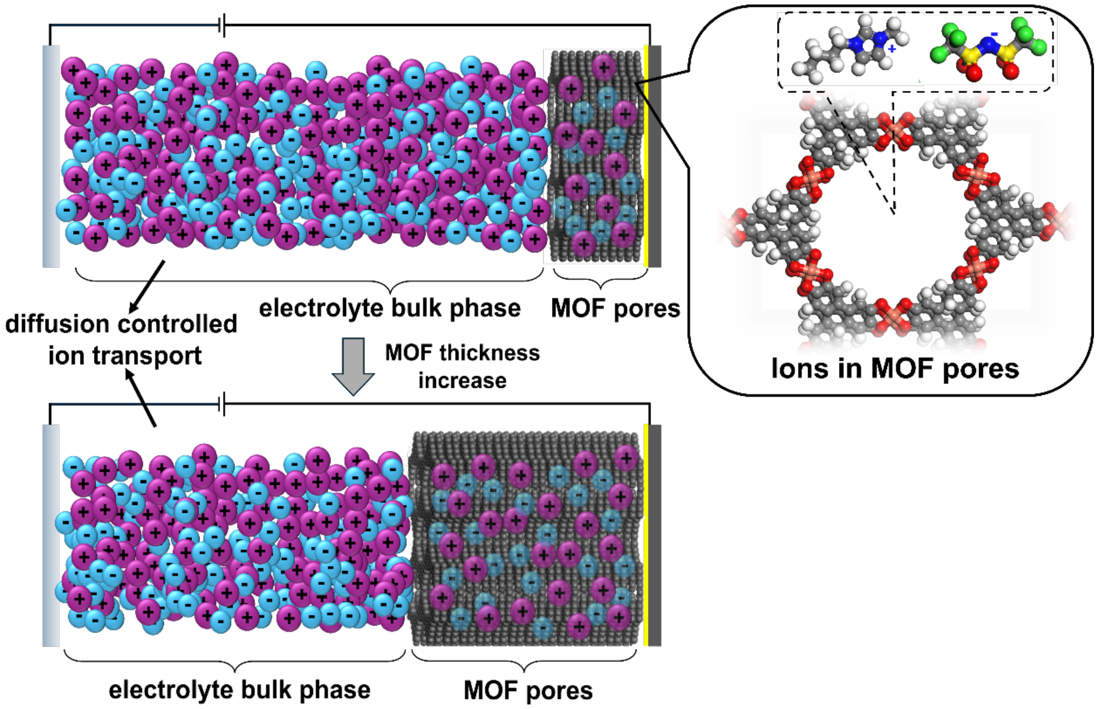

Figure S18. Sketch of ion transport in the bulk electrolyte and MOF pores.
